# Wind turbine icing characteristics and icing-induced power losses to utility-scale wind turbines

**DOI:** 10.1073/pnas.2111461118

**Published:** 2021-10-11

**Authors:** Linyue Gao, Hui Hu

**Affiliations:** ^a^Department of Aerospace Engineering, Iowa State University, Ames, IA 50011

**Keywords:** wind energy, wind turbine icing, unmanned aerial system (UAS), wind farm field campaign

## Abstract

A field campaign was carried out to investigate ice accretion features on large turbine blades (50 m in length) and to assess power output losses of utility-scale wind turbines induced by ice accretion. After a 30-h icing incident, a high-resolution digital camera carried by an unmanned aircraft system was used to capture photographs of iced turbine blades. Based on the obtained pictures of the frozen blades, the ice layer thickness accreted along the blades’ leading edges was determined quantitatively. While ice was found to accumulate over whole blade spans, outboard blades had more ice structures, with ice layers reaching up to 0.3 m thick toward the blade tips. With the turbine operating data provided by the turbines’ supervisory control and data acquisition systems, icing-induced power output losses were investigated systematically. Despite the high wind, frozen turbines were discovered to rotate substantially slower and even shut down from time to time, resulting in up to 80% of icing-induced turbine power losses during the icing event. The research presented here is a comprehensive field campaign to characterize ice accretion features on full-scaled turbine blades and systematically analyze detrimental impacts of ice accumulation on the power generation of utility-scale wind turbines. The research findings are very useful in bridging the gaps between fundamental icing physics research carried out in highly idealized laboratory settings and the realistic icing phenomena observed on utility-scale wind turbines operating in harsh natural icing conditions.

While winters are supposed to be the best season for wind energy harvesting due to the generally higher wind speeds and increased air density with the decreasing temperature, icing represents the most significant threat to the integrity and efficiency of wind turbines in cold climates (1–3). While the total installed wind power is approaching 800 GW globally, over 30% of the wind turbines are installed in the regimes of cold climates (4). About 94%, 72%, and 19% of the wind turbines were found to encounter various icing events in Europe, North America, and Asia, respectively (5).

It has been found that even a light icing event, such as frost, could produce enough surface roughness on turbine blades to reduce their aerodynamic efficiency considerably, resulting in substantial power reduction of the wind turbines (6, 7). For wind farm sites with significant ice, icing-induced power output losses are found to reach over 20% of annual energy production (4). In the case of extreme icing, it may not be possible to start wind turbines, with subsequent loss of all the possible power production for long periods of time. One notable example to highlight the importance of wind turbine icing protection is the massive turbine shutdown after a severe storm blasted Texas in February 2021. Frozen turbines were blamed as being partially responsible for the weeks-long blackout that affected millions of Texans.

While extensive investigations were conducted to study wind turbine icing physics and to develop effective anti-/de-icing systems for wind turbine icing protection, the majority of that research was conducted under idealized laboratory settings and unrealistic assumptions. For example, the wind turbines were assumed to site over flat surfaces with uniform incoming winds, work under idealized icing conditions, and maintain the same operating status (8–10). However, all wind turbines are actually installed in wind farms sited over complicated terrains and exposed to highly turbulent surface winds with airflow speed and direction varying constantly. The weather would also change dramatically during icing occurrences (e.g., snowstorms and freezing rain/drizzle). There are significant knowledge disparities between the fundamental icing physics studies performed under idealized laboratory settings and the realistic ice accretion process taking place on large, utility-scale wind turbines. Comprehesive field measurements to quantify the characteristics of ice accretion on wind turbines exposed to turbulent surface winds in realistic, natural icing environments are critical to fill the knowledge gaps to gain further insight into wind turbine icing phenomena. In this brief report we present the measurement results of a field campaign to quantify the ice accretion features over large turbine blades (50 m in length) and to assess the power losses to the utility-scale wind turbines induced by the ice accretion.

## Quantification of Ice Accretion Features on the Blades of Multimegawatt Wind Turbines

A mountainous wind farm situated near the East China Sea was selected for the present field campaign. On the farm, 31 sets of 1.5-MW wind turbines were sited along mountain ridges at altitudes ranging from 1,100 to 1,800 m above the sea level. The turbines were found to experience icing events frequently in winters, including in-cloud icing (i.e., freezing fog) and precipitation icing (i.e., wet snow or freezing rain/drizzle) since being installed in 2016.

**Fig. 1.**
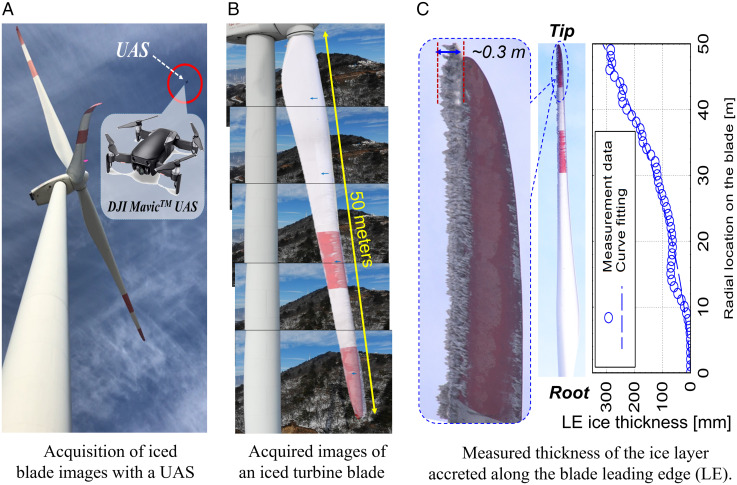
Field measurements to quantify the ice accretion on turbine blades.

For the field campaign, a high-resolution digital camera carried by an unmanned aircraft system (UAS) was used to acquire pictures of the ice structures accreted on turbine blades (Fig. 1*A*). By aligning the pictures of the blade sections properly, the ice structures accreted along the entire span of the turbine blades were recorded with reasonably good image resolution (Fig. 1*B*). Since similar ice accretion characteristics were found for all the investigated turbines, only the results taken from a preselected wind turbine are presented here for conciseness. The wind turbine has a rotor diameter of 100 m (50-m-long blades) and a hub height of 78 m. The cut-in, rated, and cut-out wind speeds of the wind turbine are 2.8 m/s, 8.9 m/s, and 18.0 m/s, respectively. During the 4-d period of the field campaign, the turbine was found to experience an icing event of 30 h in duration. The temperature measured at the hub height of the wind turbine fluctuated from −7.5 °C to −2.0 °C during the icing event and the air was found to have high relative humidity (RH) values (RH fluctuating from 75 to 100%).

By using a comprehensive image processing procedure (11), the thickness of the ice layer accreted along the leading edge of a turbine blade (LE ice thickness) was extracted quantitatively from the pictures of the iced blade taken after the icing event. While ice was observed to accrete on the entire blade span of 50 m, more ice was revealed to accrete on the outboard blade. More specifically, the LE ice thickness was discovered to increase monotonically from root to tip, with the ice thickness near the tip of the turbine blade reaching up to 0.30 m after the 30-h-long icing event (Fig. 1*C*). This is believed to be caused by the fact that, corresponding to the greater sweeping area of an element on the blade, more airborne supercooled water droplets and/or frozen raindrops would be collected on the outboard, resulting in more ice structures accreted near the blade tip than near the root of the turbine blade. A parabolic function was found to fit the measured LE ice layer thickness data quite well.

## Characterization of the Icing-Induced Power Reductions to the Wind Turbines

**Fig. 2.**
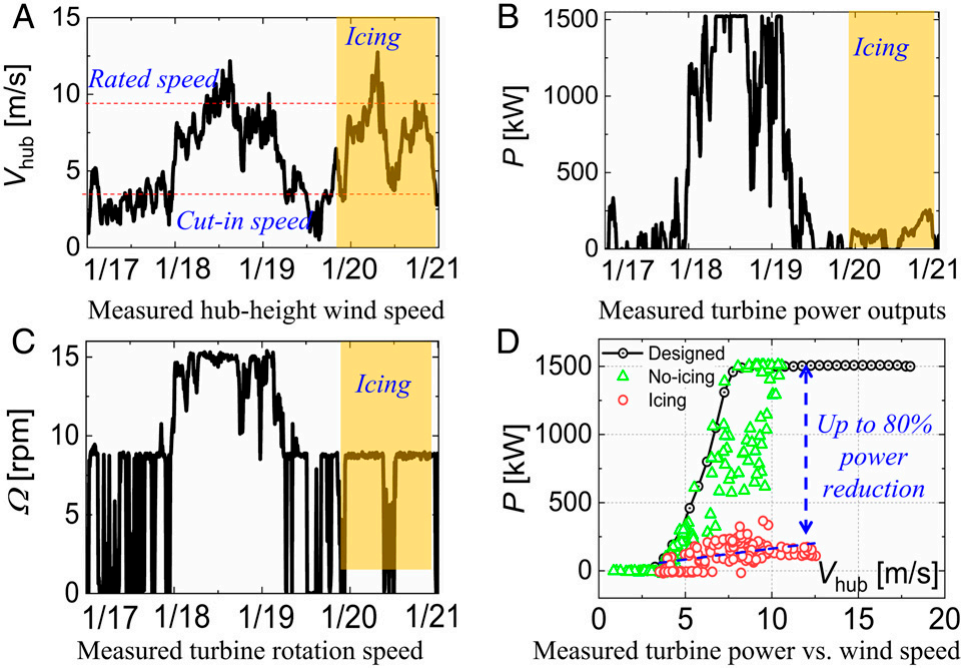
Characterization of the turbine power losses due to ice accretion.

With turbine operating data provided by the turbine’s supervisory control and data acquisition system, the detrimental effects of the ice accretion over the blade surfaces on the turbine power outputs were also investigated systematically. Even though the incoming speed of the surface winds measured at turbine’s hub height during the icing event was relatively high (substantially higher than the turbine’s cut-in speed, as indicated in Fig. 2*A*), the turbine’s power outputs were found to be quite low in general (Fig. 2*B*). This implies that the aerodynamic performance of the turbine blades was greatly degraded because of the ice accretion over the blade surfaces (6, 7), and only very limited torques could be generated during the icing occurrence, despite the high wind. Therefore, the frozen turbine was observed to rotate much more slowly, or even to stop completely from time to time (Fig. 2*C*), due to insufficient torques to keep the blade rotating. As a result, the turbine’s power outputs during the icing event were much lower in comparison to those under regular working conditions without ice accumulation on the blades.

The detrimental impacts of the ice accretion on the turbine’s power generation were revealed much more clearly by replotting the turbine power outputs as a function of the incoming wind speed (Fig. 2*D*). While the measured power outputs of the turbine were found to follow its designed power curves well in general under regular operating conditions (e.g., before the icing event), the turbine could only generate about 20% of its designed power output because of serious ice accretion on the turbine blades. Averaged power loss over the icing event (30 h in duration) was found to be up to 80%. This is extremely unfavorable because electricity would be in great demand and have the highest price during winter storms (12), which is highly likely what had happened during the Texas blackout in February 2021.

## Discussion

This field campaign quantified the ice accumulation features on multimegawatt wind turbines and systematically analyzed detrimental impacts of the ice accumulation on utility-scale wind turbine power generation. The research findings are very helpful in bridging the knowledge disparities between fundamental icing physics research with idealized settings/assumptions and the complicated icing phenomena taking place on the blades of utility-scale wind turbines as they operate in highly turbulent surface winds with ferocious, natural icing conditions. It should also be noted that since the wind farm site of the present field campaign is not far from the East China Sea, the wind turbines were exposed to high moisture and experienced a relatively wet icing process. Because of the nearby Gulf of Mexico, the wind turbines in Texas wind farms would have experienced a very similar icing scenario in the severe winter storm in Texas in February 2021. Therefore, the research findings reported here would also be very helpful to explain what happened to the wind turbines during the Texas blackout in the winter of 2021.

## Materials and Methods

The 1.5-MW wind turbines used for the field measurements are variable-speed and variable-pitch regulated turbines in an upwind arrangement. During the operation, all three blades of each wind turbine were collectively controlled to have the same pitch angles. The turbine blades have a circular-shaped cross-section profile (2.0 m in diameter) at the root to provide strong support to overcome the significant structural loads, and the cross-section profiles in the outboard region are aerodynamically optimized with different airfoil profiles (changing from DU400 to DU350, then DU300 to DU250 in the midspan, and finally from NACA63_421 and NACA63_618 in the tip region). The chord length of the blade is 2.0 m at the blade root (i.e., in the circular shape) and only about 0.2 m at the blade tip. The maximum chord length of $C_{\max}$ = 3.62 m is in the cross-section of *r*/*R* = 0.20. There are several “arrow” marks preprinted on the blades, which were utilized as the reference points to align the acquired images of the iced blade sections.

The UAS-based imaging system used for the field campaign was a DJI Mavic Air 4K equipped with a high-resolution digital camera (4,056 pixels × 3,040 pixels in resolution). A comprehensive image processing procedure, including Gaussian filtering for noise reduction and background removal, binary treatment for edge enhancement, and a Canny method for edge identification, is used to extract quantitative information from the images of iced turbine blades. Since the ice accreted on the turbine blades is highly three-dimensional with complex topological features, the outlines of the ice layers extracted from the images of the iced turbine blades represent the outmost profiles of the ice layers accreted along the leading edges of the turbine blades.

## Data Availability

The acquired images of iced blades , measured LE ice thickness, and turbine power generation data are included in the article. All the other measurement results obtained during the field campaign have been deposited at the website of the Aircraft Icing Physics and Anti-/De-Icing Technology Laboratory of Iowa State University (https://www.aere.iastate.edu/icing/).
